# Solid Platinum Nanoprobes for Highly Reliable Conductive Atomic Force Microscopy

**DOI:** 10.1021/acsami.3c01102

**Published:** 2023-04-21

**Authors:** Jonas Weber, Yue Yuan, Fabian Kühnel, Christoph Metzke, Josef Schätz, Werner Frammelsberger, Günther Benstetter, Mario Lanza

**Affiliations:** † Materials Science and Engineering Program, Physical Science and Engineering Division, King Abdullah University of Science and Technology (KAUST), Thuwal 23955-6900, Saudi Arabia; ‡ Department of Electrical Engineering and Media Technology, Deggendorf Institute of Technology, Dieter-Görlitz-Platz 1, 94469 Deggendorf, Germany; § Department of Applied Physics, University of Barcelona, Martí i Franquès 1, 08028 Barcelona, Spain; ∥ Department of Electrical Engineering and Information Technology, University of the Bundeswehr Munich, Werner-Heisenberg-Weg 39, 85577 Neubiberg, Germany; ⊥ Department of Electrical Engineering, Helmut Schmidt University/University of the Federal Armed Forces Hamburg, Holstenhofweg 85, 22043 Hamburg, Germany; # Infineon Technologies AG, Wernerwerkstraße 2, 93049 Regensburg, Germany; ∇ Chair of Electronic Devices, RWTH Aachen University, Otto-Blumenthal-Straße 2, 52074 Aachen, Germany; ○ Department of Mechanical Engineering and Mechatronics, Deggendorf Institute of Technology, Dieter-Görlitz-Platz 1, 94469 Deggendorf, Germany

**Keywords:** nanoelectronics, conductive atomic force microscopy, nanoprobe, reliability, degradation

## Abstract

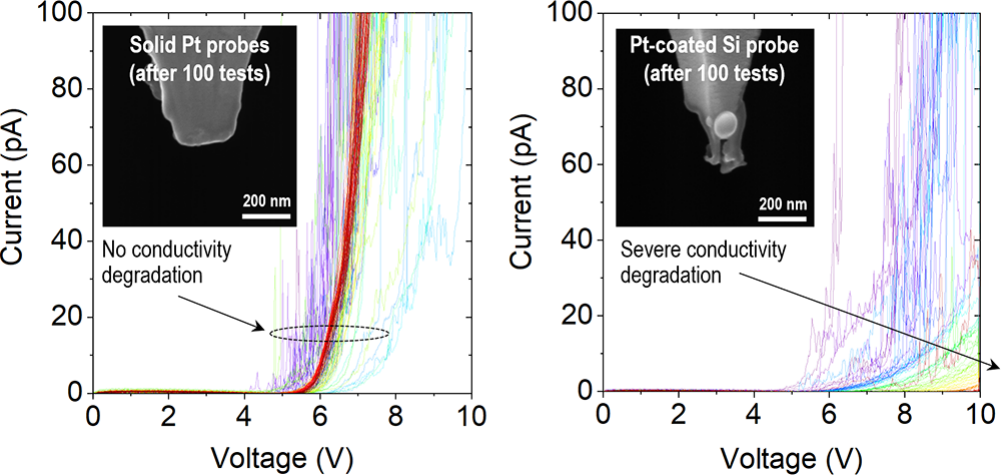

Conductive atomic force microscopy (CAFM) is a powerful technique to investigate electrical and mechanical properties of materials and devices at the nanoscale. However, its main challenge is the reliability of the probe tips and their interaction with the samples. The most common probe tips used in CAFM studies are made of Si coated with a thin (∼20 nm) film of Pt or Pt-rich alloys (such as Pt/Ir), but this can degrade fast due to high current densities (>10^2^A/cm^2^) and mechanical frictions. Si tips coated with doped diamond and solid doped diamond tips are more durable, but they are significantly more expensive and their high stiffness often damages the surface of most samples. One growing alternative is to use solid Pt tips, which have an intermediate price and are expected to be more durable than metal-coated silicon tips. However, a thorough characterization of the performance of solid Pt probes for CAFM research has never been reported. In this article, we characterize the performance of solid Pt probes for nanoelectronics research by performing various types of experiments and compare them to Pt/Ir-coated Si probes. Our results indicate that solid Pt probes exhibit a lateral resolution that is very similar to that of Pt/Ir-coated Si probes but with the big advantage of a much longer lifetime. Moreover, the probe-to-probe deviation of the electrical data collected is small. The use of solid Pt probes can help researchers to enhance the reliability of their CAFM experiments.

## 1 Introduction

Conductive atomic force microscopy (CAFM) has become a very powerful technique to analyze the topographic and electronic properties of different materials and devices with nanoscale spatial resolution.^1,2^ Its origin dates back to 1993 when Murrell et al. modified a standard AFM by coating its silicon probe with 100 nm Ti and connecting it to the positive terminal of a source meter, while connecting the negative terminal to a 12 nm-thick SiO_2_/Si sample.^3^ With this setup, the authors measured ramped voltage stresses at multiple locations in a matrix pattern with point-to-point distances of ∼20 nm and, by extracting the voltages at which tunneling occurred for each individual location, constructed the first AFM image that contained electrical information.

In the following years, major improvements were presented. To cite a few, in 1995, O’shea et al. improved the lateral resolution of the CAFM to 10 nm using enhanced probes;^4^ in, 1996 Ruskell et al. developed a dual feedback loop that monitors topography and current independently,^5^ achieving a lateral resolution of ∼8 nm; and in 1998, Olbrich et al. analyzed SiO_2_ thin films below 5 nm and reported an exponential correlation between tunneling current and thickness reduction, emphasizing the demand for precise thickness control to improve thin film reliability by avoiding local dielectric breakdown at thinner spots.^6,7^ CAFM can also apply mechanical stresses to the samples,^8^ making it ideal for a wide range of applications in the field of flexible electronics, such as characterization of piezoelectricity in two-dimensional materials^9,10^ and nanowires^11,12^ and four-dimensional characterization of materials and devices.^13,14^

CAFM employs conductive nanoprobes terminated in a small tip radius (*R*_TIP_), in most cases below 50 nm.^15,16^ The most widely used CAFM probes are made of bulk silicon, as this material is cheap and easy to sharpen using dry and wet etching techniques, which is then coated with a thin (<50 nm) Pt film to supply high conductivity^16^—the Pt film must be thin to maintain a low *R*_TIP_ in order to obtain images with a high lateral resolution. However, these probes degrade very fast due to mechanical frictions and high current densities, which generate heat and melt the metallic coating.^2,15^ To avoid this problem, one possible solution is to coat the Si probes with doped diamond^17^ or to use solid doped-diamond probes^18^ (typical dopants used are B and N).^19,20^ However, doped-diamond probes are more expensive and, more importantly, often damage the surface of the samples due to their high stiffness. Using graphene to protect the apex of metal-coated silicon probes has been proposed,^2,21^ but these probes are still not commercially available.

Using solid Pt probes is an option that in the past few years has gained more and more adepts,^22,23^ as they are durable, do not damage most samples, and their price is lower than that of doped-diamond probes (coated or solid).^21^ However, there is still no thorough statistical quantification of the performance of solid Pt probes compared to traditional Pt/Ir-coated Si probes. In this article, we statistically analyze the conductivity, resolution, and durability of solid Pt probes during CAFM measurements and under different working conditions. We find that the resolution of topographic and current maps obtained with solid Pt probes is very similar to those obtained using Pt/Ir-coated Si probes, but the solid Pt probes show the advantage of being much more durable in all kinds of experiments performed. Our study provides useful knowledge for all CAFM users that could help to enhance the reliability of their investigations.

## 2 Experimental Setup

**Figure 1:**
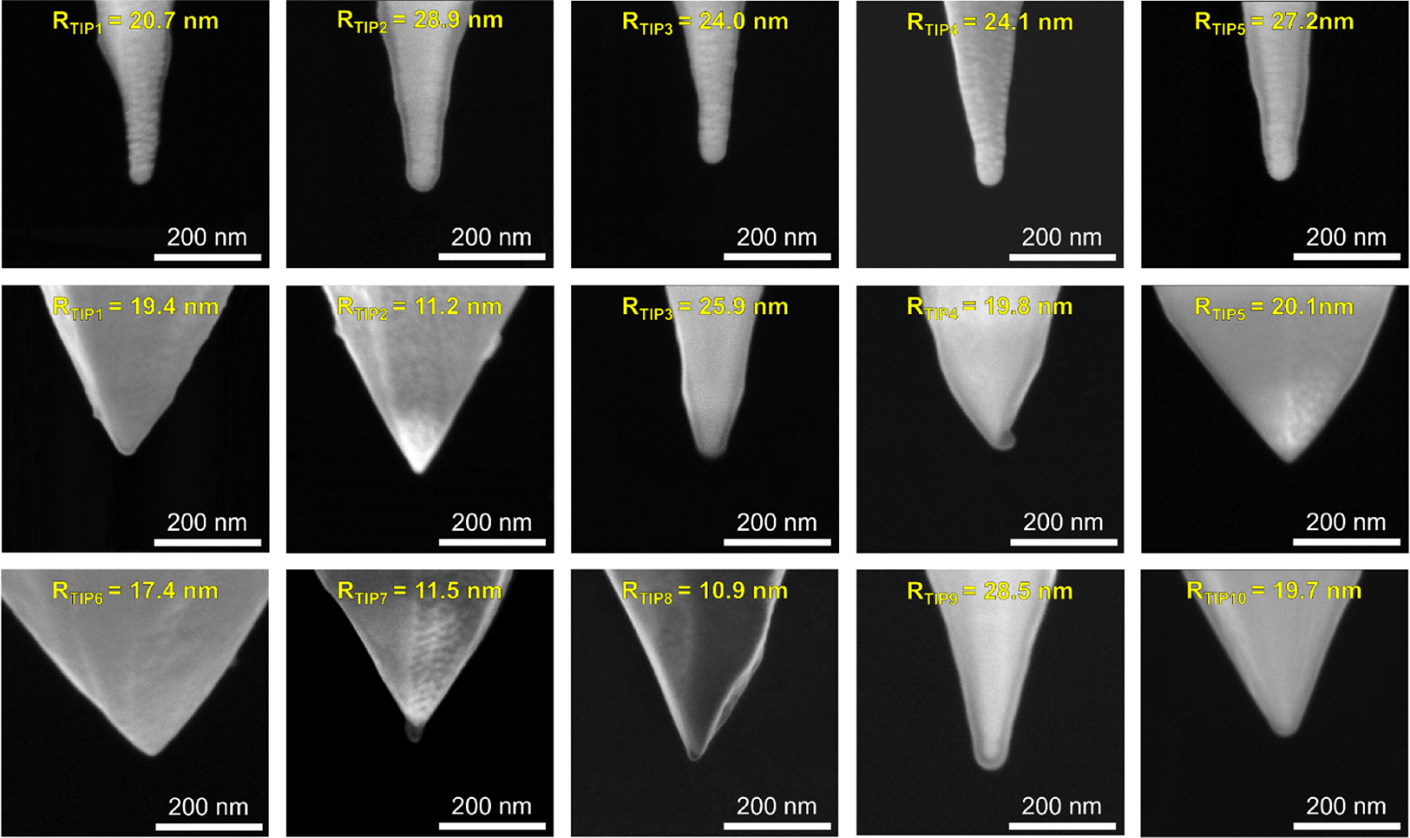
Sharpness of Pt/Ir-coated and solid Pt CAFM probes. Scanning electron images of the apex of different CAFM probes. The first row depicts Pt/Ir-coated probes (model ContV-Pt), while the second and the third rows depict solid Pt probes (model RMN-25Pt300).

In this study, we use a Dimension Icon AFM from Bruker operating in an air atmosphere (relative humidity, RH = 53.5%), which is provided with four types of probes. The first type is the NCHV-A from Bruker (nominal *R*_TIP_ = 8 nm, and *k* = 40 N/m);^24^ these are Si probes usually employed for topographic measurements in tapping mode. Note that tapping mode provides the highest resolution in topographic AFM measurements due to the small radius of the tip and the lack of lateral frictions;^25,26^ hence, the topographic measurements obtained with this type of probes are taken as a reference to evaluate the quality of the other probes. The second type is the ContV-Pt from Bruker (nominal *R*_TIP_ = 25 nm and *k* = 0.2 N/m);^27^ these are Si probes with a Pt/Ir-coating on the tip and cantilever frontside for electrical measurements in contact mode. The manufacturer of these tips did not give information about the thickness and composition of the Pt/Ir-coating, i.e., we cannot know if the term Pt/Ir refers to one layer of Pt on a Ir layer or if the Pt/Ir refers to an alloy (as used by other manufacturers,^28^ see also Table S1) and in such a case, what the proportion is. The third type is the SCM-PIT-V2 from Bruker (nominal *R*_TIP_ = 25 nm and *k* = 3 N/m);^29^ these are also Pt/Ir-coated Si probes similar to the ContV-Pt, with the main difference of a higher spring constant. Lastly, the fourth type is the RMN-25Pt300b from Rocky Mountain Nanotechnology (nominal *R*_TIP_ = 20 nm and *k* = 18 N/m);^30^ these are probes with both the tip and cantilever made from solid Pt, also for electrical measurements in contact mode. We analyze multiple ContV-Pt and RMN-25Pt300b probes using a scanning electron microscope (SEM, model Nova NanoSEM 630 from FEI) and estimate *R*_TIP_ by fitting the apex of each tip with a circle, as proposed in the reference.^31^ As depicted in Figure 1, when measuring five Pt/Ir-coated tips (with nominal *R*_TIP_ = 25 nm), we observe that the real *R*_TIP_ varies from 20.7 to 28.9 nm, and when measuring 10 solid Pt tips (with nominal *R*_TIP_ = 20 nm), we observe that the real *R*_TIP_ varies between 10.9 and 28.5 nm. We cannot know if these values fall within the specifications because none of the manufacturers indicate the variability of *R*_TIP_ in their websites. In any case, the variability of *R*_TIP_ for both types of tips seems to be reasonable and acceptable.

We perform different experiments to quantify the quality of topographic maps, current maps, and spectroscopic measurements recorded with each type of probe. To do so, we used five different samples with different properties. Samples 1, 2, and 3 consist of 3.4, 4.7, and 5.6 nm SiO_2_ grown by rapid thermal oxidation on highly conductive n-type (100) silicon (doped with arsenic, resistivity 0.0025–0.0035 Ωcm). These samples represent the state-of-the-art in SiO_2_ dielectrics, i.e., they are very homogeneous, exhibit a low concentration of local defects, and key parameters like electron mass and barrier height are well understood. This is useful to reduce the number of uncertainties in the tip/sample system and allows us to better understand what is producing the differences in the current signals registered. The use of a highly conductive substrate below the SiO_2_ is beneficial because it removes the potential that normally would fall on a standard silicon substrate.^32^ Sample 4 consists of a multilayer MoS_2_ flake produced by mechanical exfoliation and transferred on a 300 nm SiO_2_/Si wafer.^33^ This sample is ideal to test the ability of the probes to measure the thickness of a material by scanning at the edge of the flake. Also, sample 5 consists of a 10-layers-thick (i.e., ∼3.3 nm) hexagonal boron nitride (h-BN) stack grown by chemical vapor deposition on a Cu foil.^34^ This sample is rougher than the SiO_2_, which allows us to evaluate the ability of the solid Pt probes to map topographic-current correlations. Moreover, this sample contains multiple local defects, whose size can be quantified with different probes.

**Figure 2:**
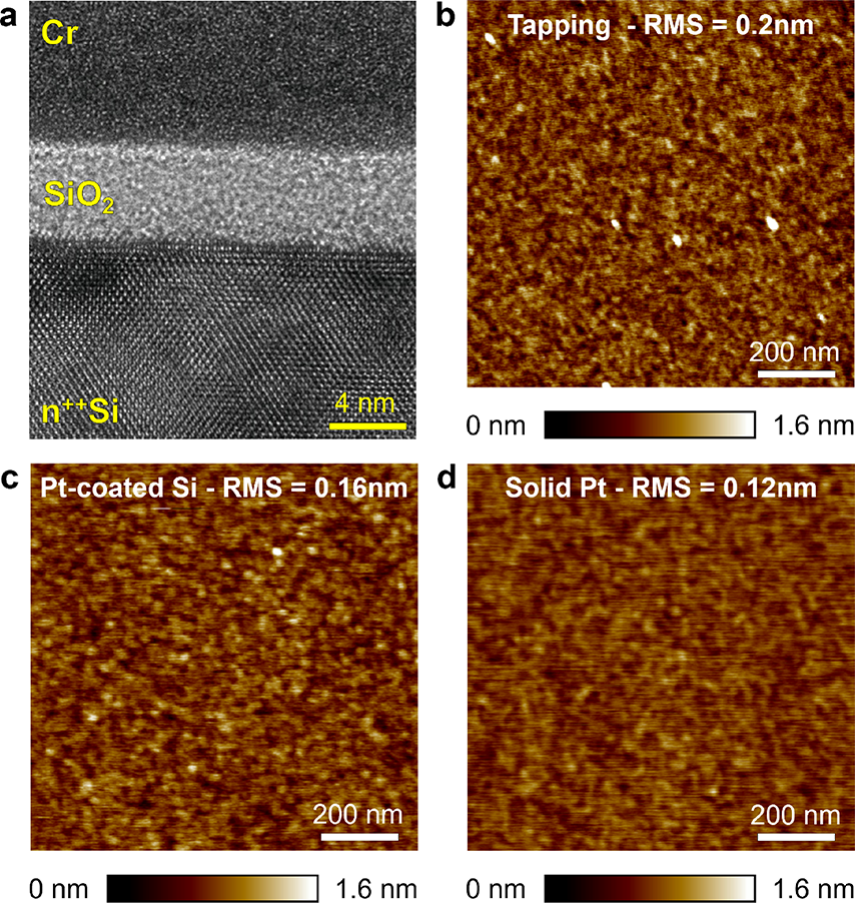
Surface roughness of a 4.7 nm SiO_2_/n^++^Si sample measured with different probes. (a) TEM image revealing the thickness and morphology of the SiO_2_/n^++^Si sample. The top Cr layer is a prerequisite for the FIB preparation prior to the TEM imaging. Topographic maps collected with (b) a Si probe (NCHV-A) in tapping mode; (c) a Pt/Ir-coated Si probe (ContV-Pt) in contact mode; and (d) a solid Pt probe (RMN-25Pt300b) in contact mode. The surface roughness of each topographic map is also indicated.

We characterize the cross-sectional morphological properties of the SiO_2_/Si samples using a transmission electron microscope (TEM, model TITAN Themis 200 from FEI), although prior to that, we had to extract a thin lamella using a focused ion beam (FIB, model Helios Nanolab 450S from FEI). The images show that the thickness of the SiO_2_ film is exactly the expected one (see Figure 2a) and that the structure of the SiO_2_ film is amorphous and homogeneous, while its surface and interface with the Si substrate are very flat/sharp. These measurements confirm the excellent quality of the industrial SiO_2_/Si samples.

## 3 Topographic Maps

First, we evaluate the topography of the SiO_2_ sample in tapping mode using a Si probe (NCHV-A). The surface of the sample is very flat, with a root mean square (RMS) roughness of 0.2 nm (see Figure 2b), in agreement with previous reports.^35^ When the topography of the same SiO_2_ sample is measured in contact mode with the Pt/Ir-coated Si probe (ContV-Pt) and the solid Pt probe, the detected RMS roughnesses of the surface are 0.16 and 0.12 nm, respectively (see Figure 2c,d). All roughness values are valid for an area of 1 by 1 μm. It is worth noting that in both cases, the topographic maps look very good but not as well-defined as in tapping mode (as expected). This observation generally could be attributed to the solid Pt probe used in this experiment having a higher *R*_TIP_ than the Si and Pt/Ir-coated Si one, which might not be following the contour of the sample so precisely (i.e., especially at cavities with high aspect ratio) leading to a flatter surface profile. However, in Figure 1 it is shown that the *R*_TIP_ of solid Pt tips is in general even slightly smaller than the one of the Pt/Ir-coated Si tips. Another possibility could be that the higher spring constant of the solid Pt tip could be improving the tip/sample contact, leading to a lower surface roughness. In any case, the differences observed are small and all tips can effectively map the surface topographic of the ultraflat SiO_2_/Si sample.

**Figure 3:**
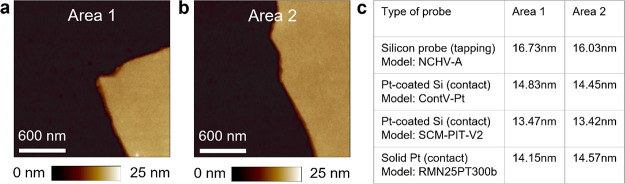
Thickness of a MoS_2_ flake measured with different probes. (a, b) Topographic maps collected with a Si probe in tapping mode at two different areas. (c) Step heights for both areas using the four different types of probes used in this study. More information is given in Figures S1–S3. The values delivered by solid Pt probes are similar to those obtained with metal-coated Si probes.

Next, we evaluate the ability of the solid Pt probes to analyze the thickness of a nanomaterial by measuring its edge; this is a typical experiment conducted in many different materials, such as two-dimensional materials,^36^ polymers,^37^ and deoxyribonucleic acid (i.e., DNA),^38^ among many others. Figure 3a,b shows the topographic maps collected with a Si probe in tapping mode at two different areas of a multilayer MoS_2_ flake produced by mechanical exfoliation and transferred on a 300 nm SiO_2_/Si wafer. We analyze the thickness of the flake detected with each probe by post-processing the images; instead of making a cross-section at a single line, we use the reliable method presented in the reference,^39^ which consists of analyzing the spectrum of the topographic images. The thicknesses of the MoS_2_ flake detected with the Si probe at areas 1 and 2 (Figure 3a,b) are 16.73 and 16.03 nm. When the experiments are repeated with the other probes, we observe that the images collected are very similar, although the thicknesses measured are slightly different (see the table in Figure 3c). In general, the conductive probes working in contact mode (ContV-Pt, SCM-PIT-V2, and RMN25Pt-300b) give thickness values 4.1 to 15.4% smaller than those obtained with the Si probes in tapping mode. A detailed discussion on the small differences observed is meaningless because (i) the differences are very small and approach to interatomic distances and (ii) the effect of the flatten or plane fit prior step edge calculation has a comparable effect; we can safely claim that the solid Pt probes delivers step edge values comparable to those obtained with Pt/Ir-coated probes, which validates their use for this type of experiment.

## 4 Current Maps

Next, we analyze the lateral resolution of the current maps collected with Pt/Ir-coated Si probes and solid Pt probes by scanning the h-BN/Cu sample. For both types of probes, we apply to the tip the minimum voltage needed to observe current above the noise level (i.e., *V*_ON_) during the scans, which is 2 V for the solid Pt probe and 6 V for the Pt/Ir-coated Si probe. This difference should be again related to the fact that the solid Pt probes have a much higher spring constant, which could produce a better (i.e., water-free) contact. Since the goal of this experiment is to check the ability of these two types of probes to map the weak spots of the h-BN sample, the scan parameters for each probe should be adjusted individually. Note that, despite many manufacturers offer Pt/Ir-coated Si probes, there is no single manufacturer that offers a model with *k* = 18 N/m (i.e., similar to the solid Pt probes used) for electrical measurements in contact mode (see Table S1); but even in the hypothetical case of having probes with identical nominal *k*, each of them should be employed with the optimum scanning parameters, which could vary from one probe to another. Hence, in our experiment, measuring with different voltages is acceptable, as far as they produce similar tunneling/leakage current across the sample under test—measuring the sample with identical scan parameters to compare the probes would be erroneous, as they would not be measuring at their optimum conditions and the currents driven would be different. We intentionally apply positive bias to the CAFM probe (with respect to the substrate) to avoid local anodic oxidation due to water splitting produced by electron injection from the tip.^40^

**Figure 4:**
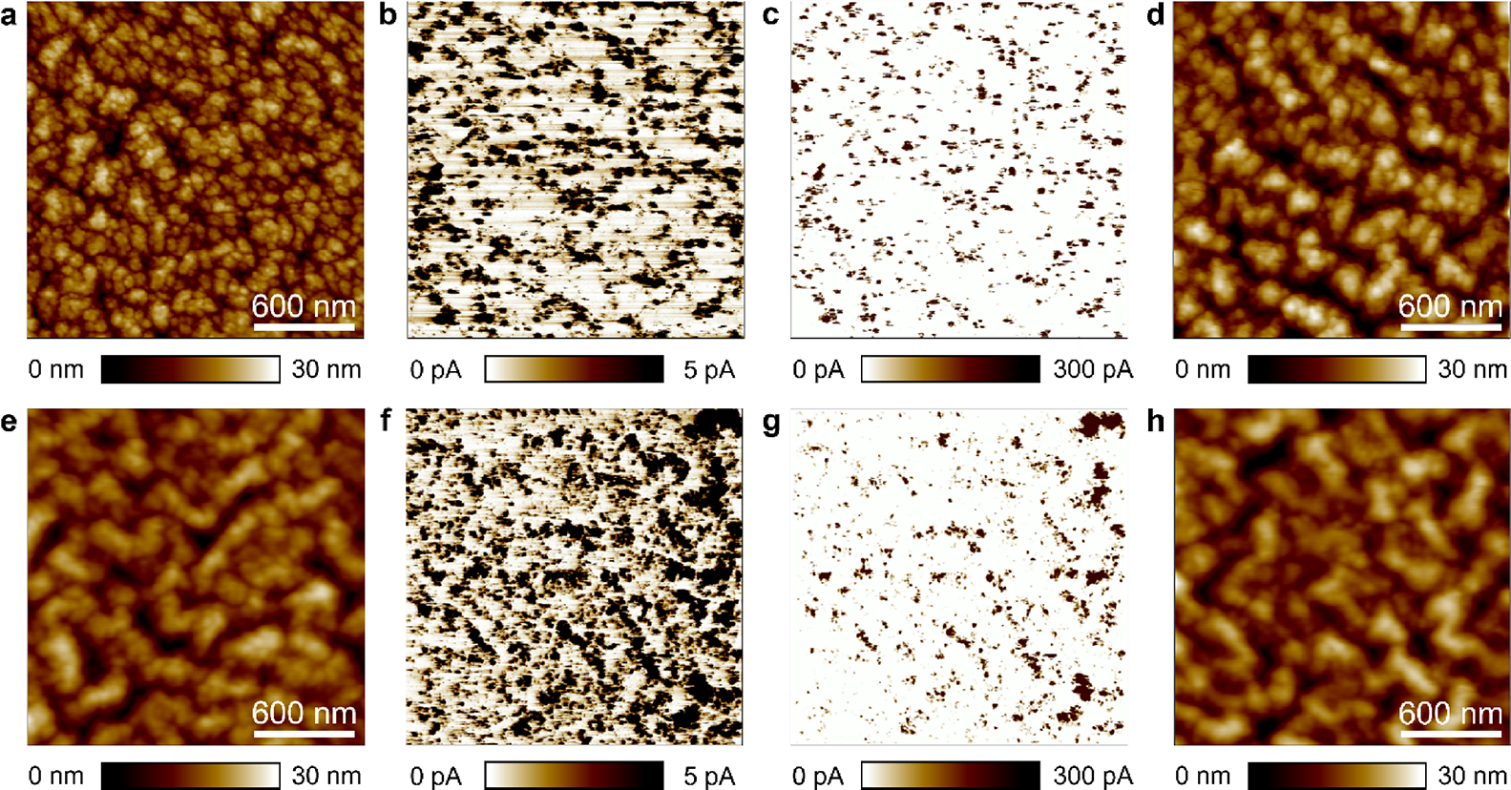
Electrical lateral resolution of Pt/Ir-coated and solid Pt probes. The data in the top row corresponds to the Pt/Ir-coated Si probes (ContV-Pt), the data in the bottom row correspond to the solid Pt probes. Topographic (a) and current (b) maps collected simultaneously on an h-BN/Cu sample using a Pt/Ir-coated probe. (c) Same current map than (b) but with a different current scale. (d) Topographic map collected on an h-BN/Cu sample using a Pt/Ir-coated probe after five subsequent current scans. The voltage applied in panels (a–d) is 6 V. Topographic (e) and current (f) maps collected simultaneously on an h-BN/Cu sample using a solid Pt probe. (g) Same current map than (f) but with a different current scale. (h) Topographic map collected on an h-BN/Cu sample using a solid Pt probe after five subsequent current scans. The voltage applied in panels (e–h) is 2 V.

The topographic maps collected with the solid Pt probes are slightly fuzzier than those collected with the Pt/Ir-coated Si probes, as it can be seen by comparing Figure 4a,e. This is in line with the observations in Figures 2 and 3, and the difference is acceptable for most types of experiments. By looking at the current maps, it can be observed that the quality of the images is very similar. At both current scales, the resolution of the probes is very similar, i.e., the size and distribution of the current spots observed with each probe are similar at both current ranges, although the currents registered with the solid Pt probe are slightly higher (see Figure 4b,c,f,g).

It is worth noting that the quality of the topographic images with the solid Pt probes is very similar as the number of scans proceeds (compare Figures 4e,h), while when using the Pt/Ir-coated Si probes, the lateral resolution reduces and some of the topographic features (i.e., cavities) that were initially detected cannot be seen anymore (compare Figure 4a,d). As the currents detected are not very high (<1 nA), the reason for this degradation must be the wearing of the tips due to mechanical friction during the scans.^15^

## 5 Spectroscopic Ramped Voltage Stresses

Next, we analyze the durability of the solid Pt probes and compare it to the Pt/Ir-coated Si probes by applying ramped voltage stresses (RVS) from 0 to 10 V to the CAFM tip (keeping the substrate grounded) at 100 randomly selected locations on the 3.4 nm SiO_2_/n^++^Si sample. This type of stress produces a very aggressive increase of current at the tip/sample junction and triggers the dielectric breakdown (DB) of the SiO_2_ film, often inducing surface epitaxy.^41,42^ To ensure that the electrical stress produced by one RVS does not affect the current registered during another RVS, we keep a safe distance of 1 μm between them.

**Figure 5:**
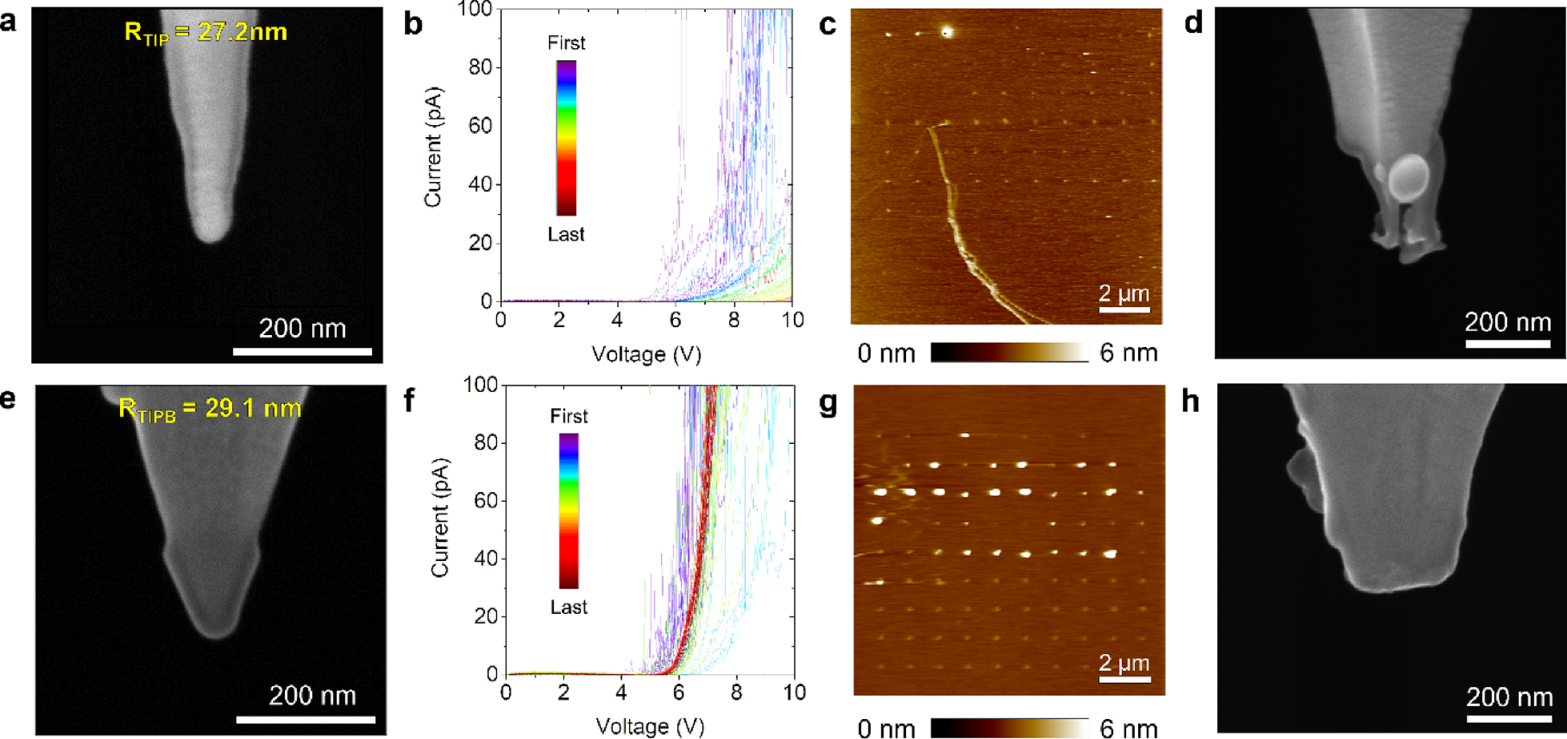
Durability of Pt/Ir-coated Si probes and solid Pt probes. The data in the top row corresponds to the Pt/Ir-coated Si probes (ContV-Pt); the data in the bottom row corresponds to the solid Pt probes. SEM images of a Pt/Ir-coated Si tip before (a) and after (d) measuring 100 *I*–*V* curves at different locations of a 3.4 nm SiO_2_/n^++^Si sample (b). A clear downward trend in the *I*–*V* curves can be observed, and the topographic AFM maps after the stress (c) indicate that at around the 50th *I*–*V* curve, the probe is unable to trigger more hard-DB events. SEM image of the Pt/Ir-coated Si tip after the stress (d) reveals that it is completely damaged by both metal coating melting (spherical particle) and Si volume removal. SEM images of a solid Pt tip before (e) and after (h) measuring 100 *I*–*V* curves at different locations of a 3.4 nm SiO_2_/n^++^Si sample (f). The topographic AFM maps after the stress (g) indicate that at almost all locations the hard-DB was triggered, as surface epitaxy can be seen. The SEM image after the scan (h) reveals that the tip has become less sharp, but it can be still used, as no degradation trend (i.e., progressive current reduction) is seen in the *I*–*V* curves (f).

First, we collect 100 *I*–*V* curves with a Pt/Ir-coated Si probe (*R*_TIP_ = 27.2 nm, see Figure 5a). The currents registered decrease as the stress proceeds, and at around the 50th *I*–*V* curve, the probe is unable to trigger hard-DB events (see Figure 5b,c). This observation evidences that the Pt/Ir-coated probe tip lost most of its conductivity during the first RVS due to the aggressive nature of the hard-DB and that the currents registered during the subsequent RVS are affected by that. This is confirmed by the dramatic tip degradation observed in SEM images after the stress (Figure 5d), which show both metallic Pt varnish melting (see spherical particle) and Si volume removal, consistent with previous studies.^2^

We then carried out the same experiment using a solid Pt probe that initially has *R*_TIP_ = 29.1 nm (see Figure 5e). The forward *I*–*V* curves (Figure 5f) show that the sample is very homogeneous, and the small random deviations of *V*_ON_ are related to the inhomogeneities of the SiO_2_/n^++^Si sample. The topographic map collected after the *I*–*V* curves (Figure 5g) shows that the hard-DB event was induced at most locations; this indicates that, despite the obvious degradation of *R*_TIP_ (see Figure 5h) the tip is still conductive, i.e., the data collected are valid and the tip can still be used to record *I*–*V* curves (although probably is not good to measure lateral scans).

## 6 Probe-to-Probe Variability

**Figure 6:**
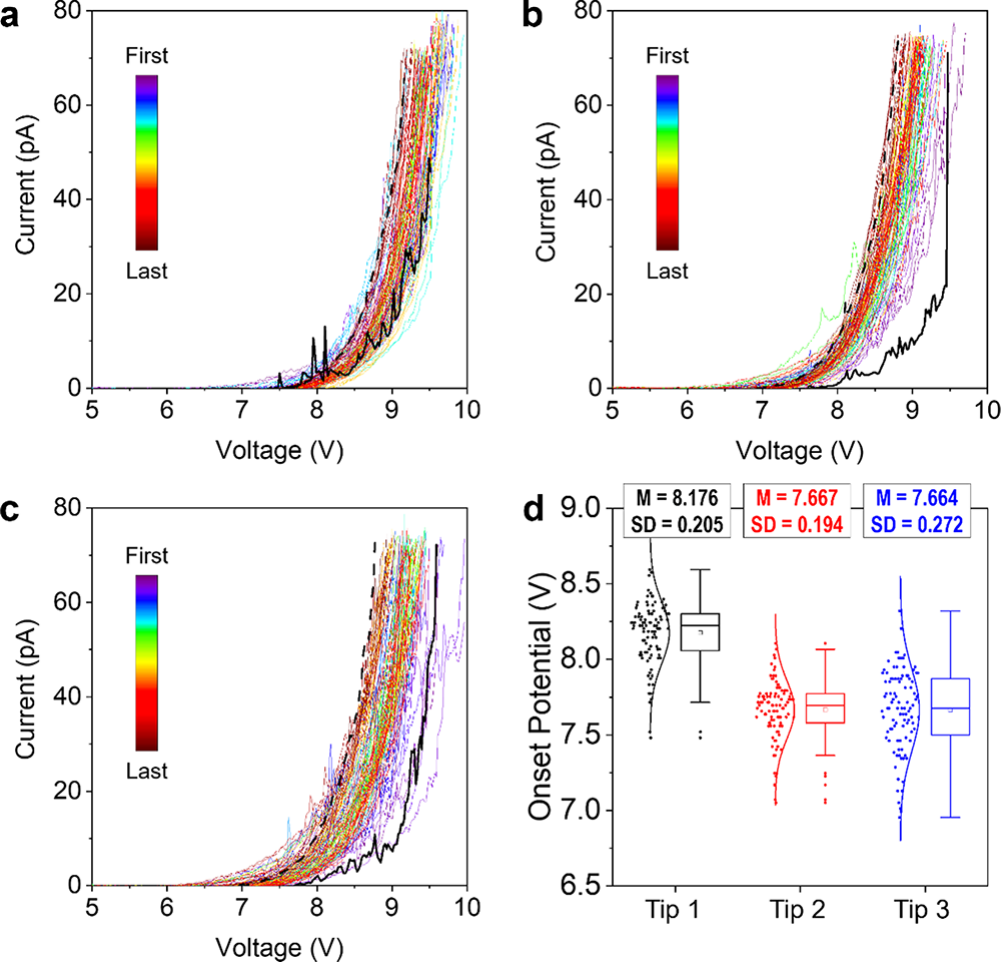
Solid Pt probe to probe variability. (a–c) Forward *I*–*V* curves recorded at 100 different locations of a 5.6 nm SiO_2_/n^++^Si sample using three different solid Pt probes (RMN-25Pt300b). The thicker black solid lines correspond to the first *I*–*V* curves, and the black dashed lines correspond to the last *I*–*V* curves; this is to show that no tip degradation (i.e., downward trend) is observed). (d) Statistical analysis of *V*_ON_ (extracted at 3pA). *M* and SD represent the mean value and the standard deviation of the distribution, respectively.

Finally, we analyze the deviation of the measurements from one probe to another by collecting 100 *I*–V curves at different locations of a 5.6 nm SiO_2_/n^++^Si sample using three different new solid Pt probes and a current limitation of 100pA. The results (see Figure 6) show that the deviation of *V*_ON_ from one probe to another is relatively small (i.e., 8.18 V ± 0.21 V, 7.68 V ± 0.19 V, and 7.66 V ± 0.27 V)—similar, if not smaller, than observed when using Pt/Ir-coated Si probes,^43^ indicating a very good reproducibility. Hence, despite the obvious and unavoidable deviation of *k* and *R*_TIP_ (which occurs in all kinds of CAFM probes), the variability of the data obtained from one solid Pt probe to another is not a concern.

## 7 Conclusions

In CAFM, the reliability of the measurements is compromised by the poor reliability of Pt/Ir-coated Si probes. Diamond-coated/solid probes are more durable, but they are much more expensive and are so stiff that they cause damage on most samples. We found that the use of solid Pt probes (*R*_TIP_ < 20 nm, *k* ≈ 18 N/m) is an excellent solution because they provide a lateral resolution that is very similar to that of Pt/Ir-coated Si probes but with the big advantage of a much longer lifetime. Moreover, the probe-to-probe variability of the electrical data collected is small. Solid Pt probes could be the solution for many scientists to improve the reliability and reduce the costs of their CAFM investigations.

## Supporting Information Available

Additional topographic maps at the edge of MoS_2_ flakes and list of various commercially available Pt-coated Si nanoprobes (PDF)
