# Supplementary material for: Solid Platinum Nanoprobes for Highly Reliable Conductive Atomic Force Microscopy
Source: ACS Appl Mater Interfaces. 2023 Apr 21;15(17):21602–8. doi: 10.1021/acsami.3c01102 (PMC10165598; doi:10.1021/acsami.3c01102)
Supplement: Supplementary file 1 — am3c01102_si_001.pdf [file am3c01102_si_001.pdf]

SUPPLEMENTARY INFORMATION

Solid Platinum Nanoprobes for Highly Reliable Conductive Atomic Force Microscopy

Jonas Weber[1-3], Yue Yuan[1], Fabian Kühnel[2,4], Christoph Metzke[2,5], Josef Schätz[6-7], Werner Frammelsberger[8], Günther Benstetter[2], Mario Lanza[1*]

[1] Materials Science and Engineering Program, Physical Science and Engineering Division, King Abdullah University of Science and Technology (KAUST), Thuwal, Saudi Arabia
[2] Department of Electrical Engineering and Media Technology, Deggendorf Institute of Technology, Dieter-Görlitz-Platz 1, 94469 Deggendorf, Germany
[3] Department of Applied Physics, University of Barcelona, Martí i Franquès 1, 08028 Barcelona, Spain
[4] Department of Electrical Engineering and Information Technology, University of the Bundeswehr Munich, Werner-Heisenberg-Weg 39, 85577 Neubiberg, Germany
[5] Department of Electrical Engineering, Helmut Schmidt University/University of the Federal Armed Forces Hamburg, Holstenhofweg 85, 22043 Hamburg, Germany
[6] Infineon Technologies AG, Wernerwerkstraße 2, 93049 Regensburg, Germany
[7] Chair of Electronic Devices, RWTH Aachen University, Otto-Blumenthal-Straße 2, 52074 Aachen, Germany
[8] Department of Mechanical Engineering and Mechatronics, Deggendorf Institute of Technology, Dieter-Görlitz-Platz 1, 94469 Deggendorf, Germany

* Corresponding author Email: mario.lanza@kaust.edu.sa

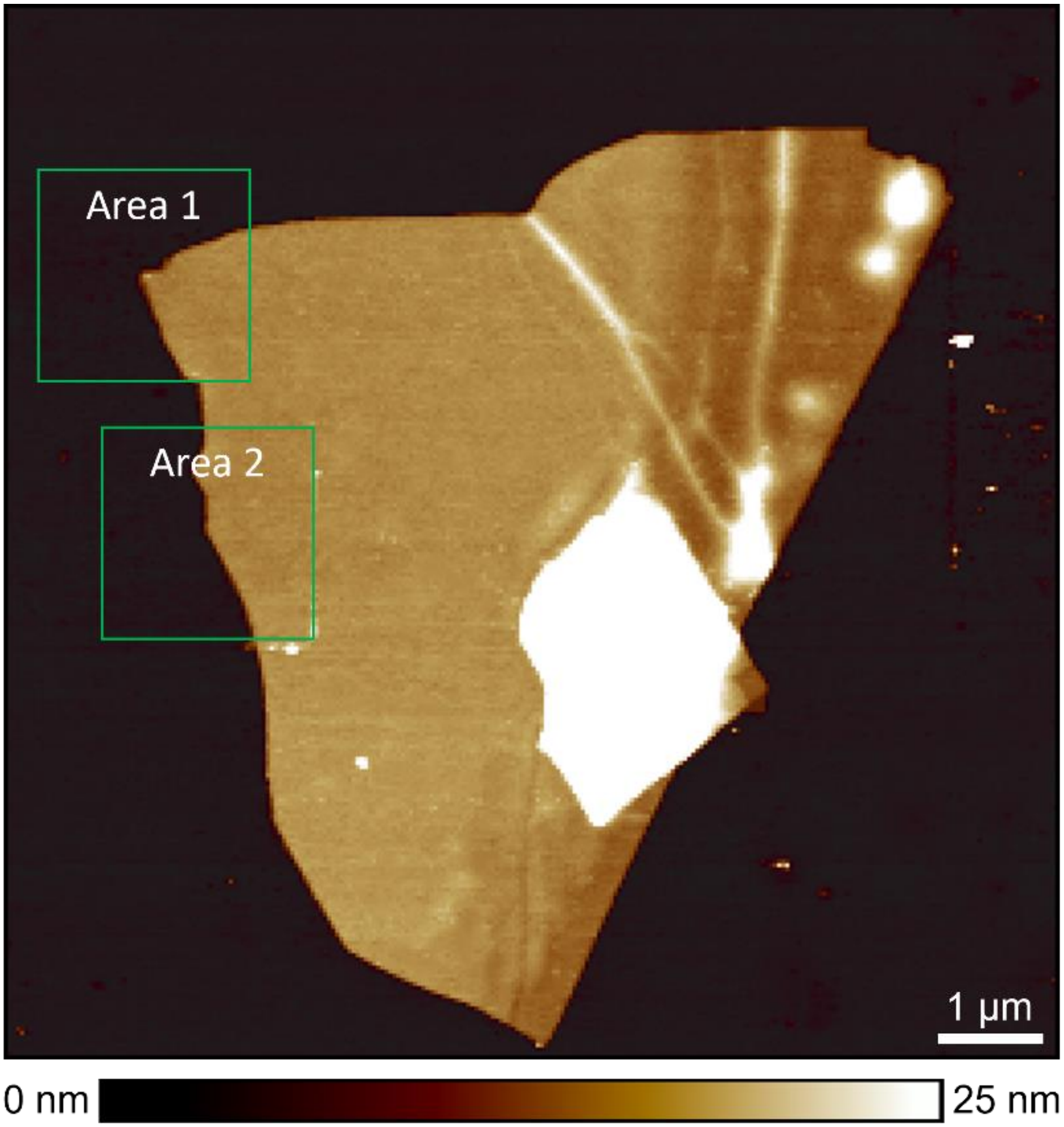

Supplementary Figure 1. Overview scan of a MoS_2 flake recorded with a NCHV-A probe in tapping mode. The locations in which the subsequent measurements were carried out are indicated in green.

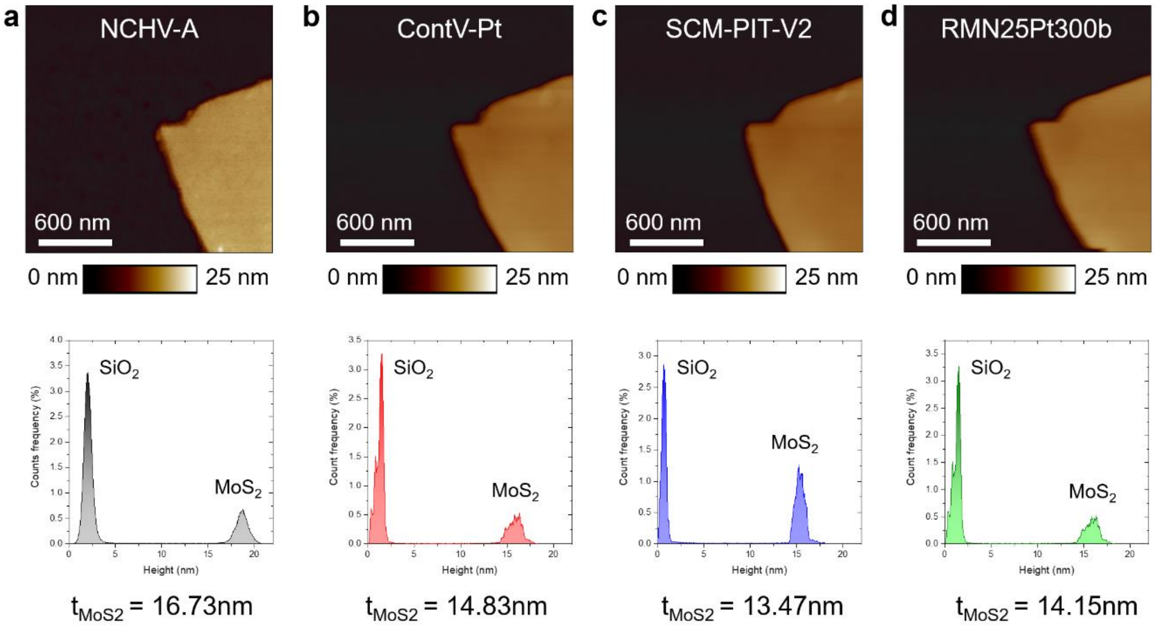

Supplementary Figure 2. Thickness measurements at location 1 of the MoS_2 flake under investigation, recorded with: (a) A Si Probe (NCHV-A) in tapping mode; (b) a Pt/Ir coated Si probe with low spring constant (ContV-Pt) in contact mode; (c) a Pt/Ir coated Si probe with intermediate spring constant (SCM-PIT-V2) in contact mode; and (d) a solid Pt probe (RMN25Pt300b) in contact mode. The histogram of each topographic map is displayed right below it. The step height t_{MoS2} is defined as the distance between the highest points of the two peaks, corresponding to the surfaces of the substrate and the MoS_2 flake.

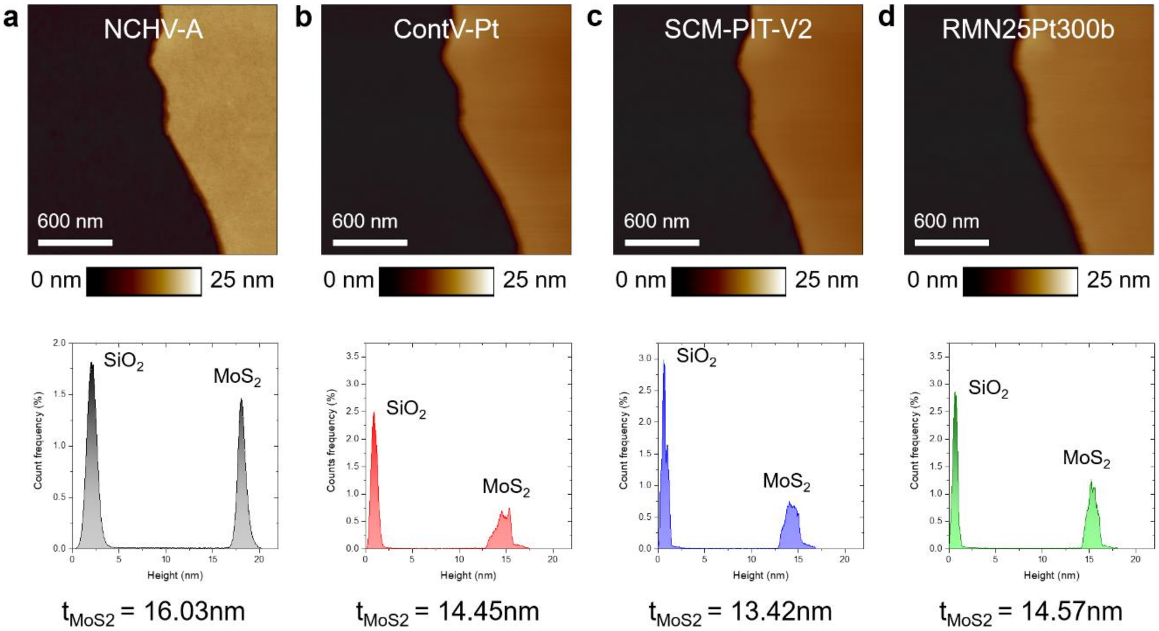

Supplementary Figure 3. Thickness measurements at location 2 of the MoS_2 flake under investigation, recorded with: (a) A Si Probe (NCHV-A) in tapping mode; (b) a Pt/Ir coated Si probe with low spring constant (ContV-Pt) in contact mode; (c) a Pt/Ir coated Si probe with intermediate spring constant (SCM-PIT-V2) in contact mode; and (d) a solid Pt probe (RMN25Pt300b) in contact mode. The histogram of each topographic map is displayed right below it. The step height t_{MoS2} is defined as the distance between the highest points of the two peaks, corresponding to the surfaces of the substrate and the MoS_2 flake.

Supplementary Table 1. List of all the commercially-available Pt-coated Si probes that we found in the internet. Despite the wide range of options, there is no supplier that offers Pt-coated Si probes for electrical measurements in contact mode with characteristics similar to those of the solid Pt probes used in this study (i.e., RMN25Pt300b, k=18N/m).

**AFM Probe supplier**	**Tip model**	**Coating**	**Recommended Mode**	**Spring constant nominal in N/m**	**Spring constant range in N/m**	**Tip radius nominal in nm**	**Tip radius range in nm**	**Price**
Appnano	ACCESS-EFM	PtIr	EFM	2.7	0.8-8.9	30	-	10 for 514.78 EUR
Appnano	ANSCM-PA	25nmPtIr +-5nm	Tapping/Non-Contact	40	25-75	30	-	10 for 396.20 EUR
Appnano	ANSCM-PA5	50nmPtIr +-5nm	Contact/Tapping/Non-Contact	40	25-45	55	-	10 for 396.20 EUR
Appnano	ANSCM-PC	25nmPtIr +-5nm	EFM	0.2	0.1-0.6	30	-	10 for 396.20 EUR
Appnano	ANSCM-PT	25nmPtIr +-5nm	EFM	3	1-5	30	-	10 for 396.20 EUR
Bruker	SCM-PIT-V2	PtIr	Contact	3	1.5-6	25	-	10 for 455.58 EUR
Bruker	CONTV-PT	PtIr	Contact	0.2	0.1-0.4	25	-	10 for 285.50 EUR
Bruker	FMV-PT	PtIr	Tapping/Non-contact	2.8	1-5	25	-	10 for 285.50 EUR
Bruker	SCM-PTSI	Platinum silicide	Contact	2.8	1-5	15	<20	10 for 1,700.08 EUR
Bruker	SCM-PIC-V2	PtIr	Contact	0.1	0.03-0.2	25	-	10 for 455.49 EUR
Bruker	PFTUNA	PtIr	PeakForceTapping	0.4	0.2-0.8	25	<35	10 for 495.98 EUR
Budgetsensors	ElectriMulti75-G	5nm Cr 25nm Pt	Contact/Tapping/Lift/FM	3	1-7	-	<25	10 for 240.00 EUR
BudgetSensors	ElectriAll-In-One Cantilever A	5nm Cr 25nm Pt	Contact	0.2	0.04-0.7	-	<25	10 for 280.00 EUR
BudgetSensors	ElectriAll-In-One Cantilever B	5nm Cr 25nm Pt	FM	2.7	0.4-10	-	<25	10 for 280.00 EUR
BudgetSensors	ElectriAll-In-One Cantilever C	5nm Cr 25nm Pt	Tapping	7.4	1-29	-	<25	10 for 280.00 EUR
BudgetSensors	ElectriAll-In-One Cantilever D	5nm Cr 25nm Pt	Tapping	40	7-160	-	<25	10 for 280.00 EUR
BudgetSensors	ElectriTap300-G	5nm Cr 25nm Pt	Contact/Tapping/Non-contact	40	20-75	-	<25	10 for 240.00 EUR
Budgetsensors	ElectriTap190-G	5nm Cr 25nm Pt	Contact/Tapping/Non-contact	48	28-75	-	<25	10 for 240.00 EUR
Budgetsensors	ElectriTap150-G	5nm Cr 25nm Pt	Contact/Tapping/Non-contact	5	1.5-15	-	<25	10 for 240.00 EUR
Budgetsensors	ElectriCont-G	5nm Cr 25nm Pt	Contact/Lateral Force	0.2	0.07-0.4	-	<25	10 for 240.00 EUR

Mikromasch	NSC18/Pt	30nm Pt	Tapping/Lift/FM	2.8	1.2-5.5	-	<30	15 for 395.00 EUR
Mikromasch	NSC35/Pt Cantilever A	30nm Pt	Tapping	8.9	2.7-24	-	<30	15 for 430.00 EUR
Mikromasch	NSC35/Pt Cantilever B	30nm Pt	Tapping	16	4.8-44	-	<30	15 for 430.00 EUR
Mikromasch	NSC35/Pt Cantilever C	30nm Pt	Tapping	5.4	1.7-14	-	<30	15 for 430.00 EUR
Mikromasch	NSC36/Pt Cantilever A	30nm Pt	FM	1	0.1-4.6	-	<30	15 for 430.00 EUR
Mikromasch	NSC36/Pt Cantilever B	30nm Pt	FM	2	0.2-9	-	<30	15 for 430.00 EUR
Mikromasch	NSC36/Pt Cantilever C	30nm Pt	FM	0.6	0.06-2.7	-	<30	15 for 430.00 EUR
Mikromasch	DPER-XSC11 Cantilever A	15nm Pt	Contact/Tapping/Lift	0.2	0.1-0.4	-	<20	15 for 490.00 EUR
Mikromasch	DPER-XSC11 Cantilever B	15nm Pt	Contact/Tapping/Lift	2.7	1.1-5.6	-	<20	15 for 490.00 EUR
Mikromasch	DPER-XSC11 Cantilever C	15nm Pt	Contact/Tapping/Lift	7	3-16	-	<20	15 for 490.00 EUR
Mikromasch	DPER-XSC11 Cantilever D	15nm Pt	Contact/Tapping/Lift	42	17-90	-	<20	15 for 490.00 EUR
Mikromasch	DPE-XSC11 Cantilever A	Pt	Contact/Tapping/Lift	0.2	0.1-0.4	-	<40	15 for 470.00 EUR
Mikromasch	DPE-XSC11 Cantilever B	Pt	Contact/Tapping/Lift	2.7	1.1-5.6	-	<40	15 for 470.00 EUR
Mikromasch	DPE-XSC11 Cantilever C	Pt	Contact/Tapping/Lift	7	3-16	-	<40	15 for 470.00 EUR
Mikromasch	DPE-XSC11 Cantilever D	Pt	Contact/Tapping/Lift	42	17-90	-	<40	15 for 470.00 EUR
Mikromasch	NSC15/Pt	30nm Pt	Tapping/Non-Contact	40	20-80	-	<30	10 for 395.00 EUR
Mikromasch	NSC16/Pt	30nm Pt	Tapping	45	30-70	-	<30	15 for 395.00 EUR
Mikromasch	NSC14/Pt	30nm Pt	Contact/Tapping	5	1.8-13	-	<30	15 for 395.00 EUR
Mikromasch	XSC11 Cantilever A	30nm Pt	Contact/Tapping/Lift	0.2	0.1-0.4	-	<30	15 for 430.00 EUR
Mikromasch	XSC11 Cantilever B	30nm Pt	Contact/Tapping/Lift	2.7	1.1-5.6	-	<30	15 for 430.00 EUR
Mikromasch	XSC11 Cantilever C	30nm Pt	Contact/Tapping/Lift	7	3-16	-	<30	15 for 430.00 EUR
Mikromasch	XSC11 Cantilever D	30nm Pt	Contact/Tapping/Lift	42	17-90	-	<30	15 for 430.00 EUR
Mikromasch	CSC37/Pt	30nm Pt	Contact	0.8	0.3-2	-	<30	15 for 430.00 EUR
Mikromasch	CSC37/Pt	30nm Pt	Contact	0.3	0.1-0.6	-	<30	15 for 430.00 EUR

Mikromasch	CSC37/Pt	30nm Pt	Contact	0.4	0.1-1	-	<30	15 for 430.00 EUR
Mikromasch	CSC17/Pt	30nm Pt	Contact/Lateral Force	0.18	0.06-0.4	-	<30	15 for 395.00 EUR
Nanosensors	PPP-CONTPt	5nm Cr 25nm Pt	Contact	0.2	0.02-0.77	-	<25	10 for 461.00 EUR
Nanosensors	PPP-CONTSCPt	5nm Cr 25nm Pt	Contact/Lateral Force	0.2	0.01-1.87	-	<25	10 for 461.00 EUR
Nanosensors	PPP-NCHPt	5nm Cr 25nm Pt	Tapping/Non-Contact	42	10-130	-	<25	10 for 461.00 EUR
Nanosensors	PPP-NCSTPt	5nm Cr 25nm Pt	Tapping/Non-Contact	7.4	1.2-29	-	<25	10 for 461.00 EUR
Nanosensors	PPP-NCLPt	5nm Cr 25nm Pt	Tapping/Non-Contact	48	21-98	-	<25	10 for 461.00 EUR
Nanosensors	PPP-EFM	25 nm double layer Cr+PtIr	EFM/Tapping/Lift	2.8	0.5-9.5	-	<25	10 for 461.00 EUR
Nanosensors	PtSi-CONT	Platinum silicide	Contact	0.2	0.02-0.77	-	<25	10 for 1,517.00 EUR
Nanosensors	PtSi-NCH	Platinum silicide	Tapping/Non-Contact	42	10-130	-	<25	10 for 1,517.00 EUR
Nanosensors	PtSi-FM	Platinum silicide	Contact/Tapping/Lift/FM	2.8	0.5-9.5	-	<25	10 for 1,517.00 EUR
Nanosensors	ATEC-CONTPt	25nm double layer Cr+PtIr	Contact	0.2	0.02-0.75	-	<20	10 for 768.00 EUR
Nanosensors	ATEC-NCPt	5nm Cr 25nm Pt	Tapping/Non-Contact	45	12-110	-	<20	10 for 768.00 EUR
Nanosensors	ATEC-EFM	25nm double layer Cr+PtIr	EFM	2.8	0.7-9	-	<20	10 for 768.00 EUR
NanoWorld	SCM-PIC	23nm PtIr	Contact	0.2	0.07-0.4	-	<25	10 for 423.00 EUR
NanoWorld	SCM-PIT	23nm PtIr	EFM/Tapping/Lift	2.8	1.2-5.5	-	<25	10 for 423.00 EUR
NanoWorld	EFM	23nm PtIr	EFM	2.8	1.2-5.5	-	<25	10 for 423.00 EUR
NanoWorld	NCHPt	23nm PtIr	Tapping/Non-Contact	42	21-78	-	<25	10 for 423.00 EUR
NanoWorld	Arrow™ EFM	23 nm PtIr	EFM	2.8	1.4-5.8	-	<25	10 for 415.00 EUR
NanoWorld	Arrow™ NCPt	23nm PtIr	Tapping/Non-Contact	42	27-80	-	<25	10 for 415.00 EUR
NanoWorld	Arrow™ CONTPt	23nm PtIr	Contact	0.2	0.06-038	-	<25	10 for 415.00 EUR
NanoWorld	CONTPt	23nm PtIr	Contact	0.2	0.07-0.4	-	<25	10 for 423.00 EUR
NanoWorld	NCLPt	23nm PtIr	Tapping/Non-Contact	48	31-71	-	<25	10 for 423.00 EUR
Nunano	Spark 150 Pt	5nmTi 40nm Pt	Tapping/Non-Contact	18	10-26	18	<30	10 for 323.98 EUR
Nunano	Spark 70 Pt	5nmTi 40nm Pt	Contact	2	0.5-3.5	18	<30	10 for 323.98 EUR
Olympus	OSCM-PT-R3	Pt	EFM	2	0.6-3.5	15	<25	10 for 656.06 EUR
OPUS	240AC-PP	25nm Pt	EFM	2	0.6-3.9	-	<25	10 for 320.00 EUR
OPUS	OSCM-PT	25nm Pt	Contact/EFM	2	0.6-3.9	-	<25	10 for 320.00 EUR

Spectrum Instruments	NSG30/Pt	25A Cr 25nm PtIr	Non-Contact	40	22-100	35	-	15 for 420 Euro
Spectrum Instruments	CSG01/Pt	25A Cr 25nm PtIr	Contact	0.03	0.003-0.13	35	-	15 for 420 Euro
Spectrum Instruments	CSG10/Pt	25A Cr 25nm PtIr	Contact	0.11	0.01-0.5	35	-	15 for 420 Euro
Spectrum Instruments	CSG30/Pt	25A Cr 25nm PtIr	Contact/Tapping	0.6	0.13-2	35	-	15 for 420 Euro
Spectrum Instruments	VIT_P_C/Pt	Pt	Contact	0.6	0.3-1	-	25-35nm	15 for 830 Euro
Spectrum Instruments	VIT_P/Pt	Pt	Non-Contact	50	25-95	-	25-35nm	15 for 830 Euro
